# Supplementary material for: The global avian invasions atlas, a database of alien bird distributions worldwide
Source: Sci Data. 2017 Mar 28;4:170041. doi: 10.1038/sdata.2017.41 (PMC5369319; doi:10.1038/sdata.2017.41)
Supplement: Supplementary File 1 [file sdata201741-s2.docx]

**Appendix A.** The mapping guidelines given to each team member for use during the mapping stage of the GAVIA project. The guidelines provide detailed instructions on how distribution maps were to be created and saved, so as to ensure consistency amongst the maps created by different team members.

**GAVIA MAPPING GUIDELINES**

**Initial set up**:

- Make a folder on your desktop called ‘GAVIA Mapping Desktop [Your initials]’ e.g. GAVIA Mapping Desktop ED.
- Copy the following layers from T:\GAVIA Project\GAVIA Maps\Basic layers into your new folder:
  - admin_GADM
  - Country_Outline_GADM
  - cities1m_vmap_Behrmann

**MAPPING**

1. Select one of your species from the spreadsheet ‘MAP MASTER SHEET’ and make a note of the SpeciesID.
2. Make a new folder within ‘GAVIA Mapping Desktop’ and call it the Latin name of whichever species you are mapping.
3. Open the GAVIA database and select the query QrySpeciesReport_[your initials]. Input the SpeciesID number for your chosen species and run the query.
4. Make a note of which records you will map and which you won’t (see notes below for rules on this), and then go back to the Distribution table (first checking to see whether anyone else is currently editing it) and change the relevant ‘Mapped Date’ and in the GISFileName column write Mapped or Not mapped. If there is no date relating to the species at all then enter the reference date into the mapped date column.
5. Rerun the query so that it includes this new information and then copy all of the data from the query and paste it into a new empty spreadsheet. Save the spreadsheet in your newly created species folder on your desktop with the name ‘[Genus species] attribute’.
6. Run a search in the folder T:\GAVIA Project\GAVIA Maps\ALL Additional DB Maps_FINAL to identify whether any maps have previously been created for your species.
7. Check the folder T:\GAVIA Project\GAVIA Maps\Ellie edits of MP maps to see if Mark Parnell has previously created a map for the species.
8. In your ‘[Genus species] attribute’ spreadsheet check the column GISFileName to see if there is a hard copy of the map in the map box.
9. Open ArcGIS and add the 3 layers listed above (cities can be turned off until needed), and any of the maps previously created for the species.
10. Create your map in a way that is appropriate. This can be either by drawing a polygon, selecting a region or island from a base layer, or by making a 10km buffer around a point. Alternatively if different distribution records are identical then you can simply make a copy of that shapefile and edit the filename and attribute table accordingly.
11. If you are using a hard copy map from the map box and it is very detailed then it can be georeferenced (i.e. you scan in the map and load it up behind your own GIS map so that you can trace it).
12. It is very important to name your shapefiles carefully and consistently. ‘Genus_species_[RecordID]_1’ is a good place to start. Then change the end of the filename to ‘_clip’ or ‘_merge’ etc. depending what you’ve just done to it.
13. Each distribution record must relate to a polygon. A single distribution record can have more than one polygon linked to it, but a single polygon can’t have more than one distribution record.
14. *Smooth* any polygons which you drew by hand (do not smooth any which were selected or clipped to a region or country).
15. If necessary *Clip* any polygons to the country layer.
16. *Merge* all of your different shapefiles together including those created from the other databases (NBN, SABAP etc.).
17. *Dissolve* all of the different polygons together so you have one line for each distribution record and so that the attribute table has only three fields:

| FID | Shape | Id |
| --- | --- | --- |

1. VERY IMPORTANT – right click on your shapefile and select *Zoom to Layer* so that you can see the full extent of your map and make sure that you haven’t accidentally selected any random countries.
2. Click on *Start Editing* and in the *Id* field copy and paste the related RecordID from your spreadsheet. Click *Stop Editing* and save edits. If you are mapping a species which has many distribution records then add *Id* numbers as you go along to avoid confusion.
3. Go back to your ‘[Genus species] attribute’ and click on the top left hand corner so that all the cells are selected, then right click and uncheck the ‘wrap text’ tick box. Then double click on one of the lines dividing the columns to that all the cells expand to fit their contents. Save the spreadsheet.
4. Go back to ArcGIS and click on *Add Data* and select your excel file ‘[Genus species] attribute’.
5. Right click on your finished layer and select *Joins and Relates*, then *Join*…
6. For 1. choose Id, for 2. the excel file and for 3. RecordID. Then click OK.
7. Open the attribute table of your finished layer to check that the attribute data has joined correctly and that the right information relates to the appropriate polygon.
8. If everything is as it should be then right click on the finished layer and select *Data – Export data*… Ensure that *Export All Features* is selected and then save it in your species folder on your desktop as ‘GAVIA_Genus_species_EXPORT’
9. Copy and Paste the entire species folder from your desktop into T:\GAVIA Project\GAVIA Maps\_Not final.
10. Then using Arc Catalog copy and paste your ‘GAVIA_Genus_species_EXPORT’ shapefile into T:\GAVIA Project\GAVIA Maps\_FINAL and rename it ‘GAVIA_Genus_species’.
11. Make a note of how many distribution records you created (not including those ‘not mapped’) so that you can keep a running tally on the MAP QUOTAS spreadsheet.

**SPLITTING**

NOTE: Anything changed in the ‘Distributions’ table of the GAVIA database cannot be undone. By editing records in this way you are essentially editing the raw base data. Therefore I cannot emphasise strongly enough how careful you need to be - please tell me *immediately* if you think you have made an error.

1. Open the GAVIA database and select the query QrySpeciesReport_[your initials]. Input the SpeciesID number for your chosen species and run the query.
2. Copy all of the data from the query and paste it into a new empty spreadsheet. Save the spreadsheet in your newly created species folder on your desktop with the name ‘[Genus species] attribute’.
3. Filter the Notes column in ascending order and highlight the records which need splitting in yellow. Any record that contains more than one date needs to be split. For example:

| Introd date | Grouped date | Mapped date | Notes |
| --- | --- | --- | --- |
| 1870s | 1875 |  | 1870s: 90+ birds released in Wellington and Hawke's Bay.  1890: abundant around Hawke's Bay, in Napier and Tutira.  1912: plentiful at Tuparoa  1922: spread up coast from Wellington to Wanganui and New Plymouth |

1. Make a note of the RecordID for your first record which needs splitting, then check that no-one else has the Distributions table of the GAVIA database open. If they don’t then you can open it.
2. Make sure the Distributions table is sorted by the RecordID and scroll down to your relevant RecordID number.
3. Enter the Mapping Date as the first date on the record (should be the same as the Grouped date).

| Introd date | Grouped date | Mapped date | Notes |
| --- | --- | --- | --- |
| 1870s | 1875 | **1875** | 1870s: 90+ birds released in Wellington and Hawke's Bay.  1890: abundant around Hawke's Bay, in Napier and Tutira.  1912: plentiful at Tuparoa  1922: spread up coast from Wellington to Wanganui and New Plymouth |

1. Then open the Office Clipboard and copy the entire record (make sure it has definitely copied).
2. Then delete out the information from the record that relates to any date after the first:

| Introd date | Grouped date | Mapped date | Notes |
| --- | --- | --- | --- |
| 1870s | 1875 | 1875 | 1870s: 90+ birds released in Wellington and Hawke's Bay.  1890: abundant around Hawke's Bay, in Napier and Tutira.  1912: plentiful at Tuparoa  1922: spread up coast from Wellington to Wanganui and New Plymouth |

1. Scroll down to the bottom of the Distribution table and paste in as many copies of the record as you will need in total. So for the example record we would need to paste in three copies.
2. Edit the copies to reflect the expansion of the species over time, **make sure you change the mapping date as well as the notes column**. DO NOT change the status column, leave that the same throughout.

| Introd date | Grouped date | Mapped date | Notes |
| --- | --- | --- | --- |
| 1870s | 1875 | 1875 | 1870s: 90+ birds released in Wellington and Hawke's Bay.  1890: abundant around Hawke's Bay, in Napier and Tutira.  1912: plentiful at Tuparoa  1922: spread up coast from Wellington to Wanganui and New Plymouth |
| 1870s | 1875 | **1890** | 1870s: 90+ birds released in Wellington and Hawke's Bay.  1890: abundant around Hawke's Bay, in Napier and Tutira.  1912: plentiful at Tuparoa  1922: spread up coast from Wellington to Wanganui and New Plymouth |
| 1870s | 1875 | **1912** | 1870s: 90+ birds released in Wellington and Hawke's Bay.  1890: abundant around Hawke's Bay, in Napier and Tutira.  1912: plentiful at Tuparoa  1922: spread up coast from Wellington to Wanganui and New Plymouth |
| 1870s | 1875 | **1922** | 1870s: 90+ birds released in Wellington and Hawke's Bay.  1890: abundant around Hawke's Bay, in Napier and Tutira.  1912: plentiful at Tuparoa  1922: spread up coast from Wellington to Wanganui and New Plymouth |

1. Have a read over your edits and make sure everything is as it should be, then *save* the Distribution table.
2. Now go back to your original spreadsheet and take off the yellow highlighted colour for that record.
3. Move onto the next record that needs splitting and repeat the process.
4. Once you have split all of the records for that species, rerun the original query (as per step 1) and then copy and paste this new set of records into a new spreadsheet (you can delete your original spreadsheet).
5. Filter the Notes column into ascending order so that you can check that everything is split correctly.
6. Map as per usual. When mapping a split record map the earliest date first. Then with the next record make a copy of the first map and expand it to cover the new regions. Continue this for all split records, so that you end up with a series of maps showing the distribution expanding over time.
7. Even if the split records do not provide any additional area data still map each one as it is important to know that the species was still present in that location at a later date. This is the same for if the species has died out or is unsuccessful, still map the region for the date that it died out as we need the record to show that it was in that specific area.

**WHEN TO MAP AND WHEN NOT TO MAP**

We want to use the most specific information available to us so that we can produce the most precise maps.

1. If the record only goes down to Country level then mark it as ‘Not mapped’ in the GISFileName box. Exceptions to this rule are if the country is particularly small (e.g. Singapore, Hong Kong), if it is a small island (e.g. any of the Pacific islands), or if it is Taiwan (we are mapping all Taiwan records, but if it is to country level then make a note of this in the Notes column of the spreadsheet).
2. If the detail goes down to an administrative district level (e.g. a county in England) then that is okay to make, but for example the states of the US would be at too large a scale. Always ask me if you are not sure.
3. Do not map a record if it is obviously a single escapee record, or just an occasional vagrant.
4. Mark it on the MASTER MAP SHEET spreadsheet if you have not mapped any of the records at all for a particular species. In the column ‘Completely not mapped’ write *Yes* and in the notes box explain why. For example ‘Only detailed to US state level’.
